# Improved Skip-Gram Based on Graph Structure Information

**DOI:** 10.3390/s23146527

**Published:** 2023-07-19

**Authors:** Xiaojie Wang, Haijun Zhao, Huayue Chen

**Affiliations:** School of Computer Science, China West Normal University, Nanchong 637002, China

**Keywords:** graph embedding, interpretability, graph structure, Skip-gram, node feature fusion

## Abstract

Applying the Skip-gram to graph representation learning has become a widely researched topic in recent years. Prior works usually focus on the migration application of the Skip-gram model, while Skip-gram in graph representation learning, initially applied to word embedding, is left insufficiently explored. To compensate for the shortcoming, we analyze the difference between word embedding and graph embedding and reveal the principle of graph representation learning through a case study to explain the essential idea of graph embedding intuitively. Through the case study and in-depth understanding of graph embeddings, we propose Graph Skip-gram, an extension of the Skip-gram model using graph structure information. Graph Skip-gram can be combined with a variety of algorithms for excellent adaptability. Inspired by word embeddings in natural language processing, we design a novel feature fusion algorithm to fuse node vectors based on node vector similarity. We fully articulate the ideas of our approach on a small network and provide extensive experimental comparisons, including multiple classification tasks and link prediction tasks, demonstrating that our proposed approach is more applicable to graph representation learning.

## 1. Introduction

As the application of graph representation learning in the real world continues, an increasing number of networks without node labels/attributes are emerging. However, these networks are challenging to configure node labels/attributes, such as online shopping platform networks and wireless sensor networks for interpersonal communication involving personal private information. Learning node features in networks with only node link information are receiving increasing attention.

Word2vec [1] is a language model in natural language processing (NLP), including the CBOW (Continuous Bag of Words) [1] model and the Skip-gram [1] model, learning two sets of vector representations of each word from the corpus by unsupervised learning. Inspired by word vector representations, Skip-gram has gained widespread attention for migrating applications in graph representation learning. With the publication of representative research results, such as DeepWalk and node2vec, researchers have developed dozens of models based on Skip-gram with different architectures in just a few years. These creative studies demonstrate that word2vec is a landmark work.

However, despite so much relevant work in graph embedding, existing studies based on the Skip-gram model do not consider the differences between graph embedding and word embedding. In word embedding, the words around the central word (within the window) are sampled, forming a training pair with the central word. Similar to word embedding, graph embedding generates training node pairs through random walks between nodes. However, in the generation of the training set by random walks and the migration application of the Skip-gram model, the degree of nodes and the shortest path length between nodes are not considered. In large corpora, the most common words (e.g., “in”, “the”, and “a”) often do not provide as much information value as the rare words [2]. However, the most common nodes in the network are those containing more connected edges (expressed by node degree), are located at the center of the network, and are crucial for learning node feature representations, as we show in the subsequent application section. For example, the nodes around the hub nodes are more likely to belong to the same class. In generating training node pairs by random walks, the location information between node pairs is ambiguous and does not fully reflect the location relationship between nodes. The existence of these differences means the Skip-gram model only partially uses the structural information implied in the network.

Compared with word embedding in NLP, graph embedding has more application scenarios, such as the Internet of Things (IoT) [3], social networks [4], and traffic forecasting [5]. In recent years, many application scenarios have required machine learning algorithms to assist in decision-making, it is an urgent need to “explain” why they obtained this result and how they proceed, leading to an increased focus on graph representation learning in interpretability studies. Therefore, interpretability studies on the Skip-gram model are very popular, such as word2vec Explained [6], NetMF [7], MCNS [8], and other related works that have theoretically investigated Skip-gram models in depth.

Although there are many works for learning node feature representation based on the Skip-gram model, no work intuitively explains the reasons for high-quality node feature generation. Visualizing the workflow of an algorithm through a case study can be very helpful in understanding the nature of the algorithmic idea [9]. It is essential to deepen researchers’ understanding of Skip-gram through a simple case study.

In the NLP field, the difference between input and output embedding of words has gained a deeper understanding. By default, word2vec discards output embeddings at the end of the training, leaving only input embeddings. In word representation learning using the Skip-gram model, the input embedding is slightly better than the output embedding [10], and the input and output embedding are similar. By visual analysis of the feature representation in the cases and the experimental results, DESM [11] shows that combining input and output embeddings in an information retrieval task outperforms using input embeddings alone.

Inspired by research on input–output embeddings of the word, we consider whether graph embedding have similar properties and whether higher-quality node features can be gained after training.

To alleviate these problems, we first provide an in-depth study of the principle for learning node feature representations based on the Skip-gram model. Through a case study, we show the process of learning node feature representations by the Skip-gram model, explaining the reasons for learning high-quality node feature representations. Benefit from the structural properties of the graph and the scalability of the Skip-gram model, we propose a Graph Skip-gram model more suitable for graph embedding; inspired by the research related to word embedding, we design a feature fusion algorithm to obtain higher quality node feature representations based on the existing two sets of node embeddings.

Specifically, our contributions are the following:

- We use our insights in combination with Skip-gram to propose Graph Skip-gram for graph embedding, which can capture the local and global information of the graph.
- We propose a novel node feature fusion algorithm: selectively fuse the two sets of feature representations generated by the graph embedding.
- Through case studies, we explore the principle of graph embedding and then visualize, analyze, and evaluate our proposed algorithm.
- We evaluate our model on multi-label classification tasks and link prediction tasks with multiple methods and datasets. Experimental results demonstrate that Graph Skip-gram learns the structural properties of the network, and our proposed feature fusion algorithm can effectively improve the quality of node embedding.

The rest of the paper is organized as follows. In Section 2, we discuss related work. In Section 3, we explain the definitions commonly used in this paper. In Section 4, we propose the Graph Skip-gram model and a new feature fusion algorithm. In Section 5, we introduce the application of the algorithm in real-world networks. Firstly, we analyze the process of generating node feature representations via graph embedding; secondly, we briefly describe the idea of our algorithm. In Section 6, we evaluate the Graph Skip-gram model through experiments with multiple methods on multiple datasets. Finally, we conclude our work in Section 7.

## 2. Related Work

### 2.1. Graph Embedding and Sensor Networks

Ref. [12] proposes a graph embedding method for solving sensor localization problems using signal strength. GEPM [13] proposes a polynomial mapping-based graph embedding method for localizing unknown nodes in wireless sensor networks. ESTNet [14] proposes an embedded spatial-temporal network using captured sensor information and stacking multiple three-dimensional convolutional units for modeling. GDN [15] proposes a combined structural learning and graph neural network approach to capture inter-sensor relationships for abnormal event detection. G-HAM [16] proposes a hierarchical attention model for human intention recognition, using graph structure to represent spatial information from electroencephalographic (EEG)-based sensors. MOAGE [17] proposes a combined outlook attention and graph embedding approach for traffic prediction tasks.

Graph embedding is widely used in sensor networks and has been used in practical applications in IoT, such as traffic signal control.

### 2.2. Graph Embedding Based on Skip-Gram Model

DeepWalk [18] uses random walks strategy to sample the target nodes, using the sequence of generated nodes as input to the Skip-gram model. Node2vec [19] designs a flexible and biased random walks strategy based on DeepWalk, integrating BFS (breadth-first search) and DFS (depth-first search) into the random walks process. Walklets [20] optimize DeepWalk’s sampling strategy on large graphs to capture multiple hierarchical relationships of nodes in the network. DP-Worker [21] proposed a degree penalty principle, relating the probability of random walks to the node degree. This category of methods focuses on the study of random walks strategies.

Struct2vec [22] defines vertex similarity in terms of spatial structural similarity. LINE [23] can be viewed as an algorithm for building neighborhoods using BFS. Using the Skip-gram model, Splitter [24] generates multiple vector representations for each node, representing a distinct hierarchical relationship within the community network. SPINE [25] proposed a biased Skip-gram negative sampling method, exploiting the structural similarity between nodes to guide a new positive sampling strategy. Edge2vec [26] embeds the edges in the network by combining deep self-encoders and the Skip-gram model through deep neural networks. CENA [27] proposes a framework for both link prediction and network alignment, utilizing the Skip-gram model and negative sampling techniques to optimize the objective function.

All the above works use the Skip-gram model to learn node feature representations. However, they are insufficiently aware difference between graph and word embedding. We recognize the differences between the two application scenarios and propose Graph Skip-gram for graph embedding.

### 2.3. Graph Embedding with Matrix Factorization

NetSMF [28] proposes a sparse matrix factorization algorithm for large-scale network embedding, improving the efficiency of graph embedding learning. GraRep [29] uses matrix factorization to solve the network embedding problem, integrating global network structure information while learning the network representation. TADW [30] proves that DeepWalk can be represented through matrix decomposition and proposes a network learning method combining textual information. Implicit SVD [31] devises a framework for computing singular value decomposition of implicitly defined matrices. AGNMF-AN [32] proposed an enhanced graph regularized non-negative matrix factorization method based on attribute networks for community detection.

However, they suffer from high computing power requirements and low performance and are difficult to implement on large networks.

### 2.4. Theoretical Analysis—Interpretability

Many works have theoretically explained Skip-gram-based graph embedding. For example, SPPMI [33] analyzes the Skip-gram model with negative sampling and shows that it implicitly factorizes a word-context matrix. Watch Your Step [34] proposes a method for automatically selecting parameters for graph embedding models to suit different networks, proving that the DeepWalk learning process is equivalent to matrix decomposition. NetMF [35] proved all negative sampling based [18,19,23,36] can be unified into a matrix factorization framework with closed forms. MCNS [8] theoretically proves negative sampling is equally significant to positive sampling in optimizing the objective function and reducing the variance, proposing a self-contrast approximation to replace the negative sampling strategy.

Although many works have theoretically investigated the application of Skip-gram to graph embedding, none have intuitively explained the reason for node feature generation.

### 2.5. Deep Learning

In graph representation learning, graph convolutional networks, such as GCN [37], GAT [38], SGC [39], GNN [40], and GraphHeat [41], are widely accepted as mainstream methods. However, their training process often requires additional node attribute information, and training is usually completed under supervised conditions, which is difficult to use in networks without node attributes/labels.

### 2.6. Feature Fusion in Sensor Networks

CFA [42] proposes a feature selection method based on performance and diversity between two features for detecting stress under certain driving conditions. FGF [43] proposes a feature graph fusion method for robot recognition using RGB and depth information collected by sensors. Grad-CAM++ [44] proposes a feature fusion technique for tool wear monitoring using hierarchical neural network by collecting vibration and sound signals. In [45], Deng et al. proposes a feature selection method based on separability and dissimilarity to enhance odor identification in gas sensor arrays. In [46], Gravina et al. survey discusses current data fusion techniques in body sensor networks and designs a generalized framework for comparison.

The above works have used different techniques to fuse sensor network node information in different scenarios. In this paper, we propose a new idea of feature fusion, using the feature similarity of the network nodes to selectively fuse node information.

### 2.7. Work in Other Directions

ComE [47] learns node embeddings based on DeepWalk and optimizes them for community detection. GraphSAGE [48] proposes a framework for inductive learning that generates unknown vertex embeddings using attribute information of vertices. WCN [49] proposes a link prediction method based on common neighbors and different types of centrality measures. MS-RPNet [50] proposes a hyperspectral image classification network that hierarchically extracts different spatial information superimposed into multi-scale spatial features. SCAE-MT [51] designs a stacked convolutional self-coding network model to extract deep features of hyperspectral remote sensing images. Some studies, such as SAE [52], DNR [53], and SDNE [54], use autoencoder to embed the network representation. However, these works do not resolve our concerns.

## 3. Preliminaries

### 3.1. Notations

Let G=(V,E) be a given network, it can be any (un)directed (un)weighted or sensor network, where *V* represents the members of the network and *E* their connections, E⊆(V×V). Let $f:V\to R^{d}$ be the mapping function from nodes to feature representations. Here, *d* is a parameter that specifies the dimension of node feature representation. In practice, *f* is a matrix of size |V|×d parameters. Let **D** denote the degree matrix, where $D\in R^{d}$, **P** denote the node distance matrix, where $P\in R^{d\times d}$, $P_{un}$ is the shortest path length between node *u* and node *n* ($P_{uu}=0$). For every source node u∈V, we define $\;N_{s}\left(u\right)\subset V$ as a network neighborhood of node *u*, generating through a neighborhood sampling strategy *S*. Table 1 summarizes the notations and abbreviations frequently used in this paper.

### 3.2. Skip-Gram Model

Given a sequence of words to be used for model training $\;w_{1},\;w_{2},\;w_{3}\ldots \;w_{T}$. The objectives of the Skip-gram model is as follows

(1) $$\frac{1}{T}\sum_{t=1}^{T}\sum_{-c\leq j\leq c,j\neq 0}\log p\left(w_{t+j}|w_{t}\right).$$

where *w* is the word in the given sentence, *c* is the size of the training context. The basic Skip-gram formulation defines $p\left(w_{t+j}|w_{t}\right)$ using the softmax function:   

(2) $$p\left(w_{O}|w_{I}\right)=\frac{\exp({v_{w_{O}}^{{}^{\prime }}}^{⊤}v_{w_{I}})}{\sum_{w=1}^{W}\exp({v_{w}^{{}^{\prime }}}^{⊤}v_{w_{I}})}.$$

Optimizing the above equation using the negative sampling technique, the objective in Equation (2) simplifies as

(3) $$\log P\left(w_{O}|w_{I}\right)=\log\sigma \left(v{{}_{w_{O}}^{\prime }}^{⊤}v_{w_{I}}\right)+\sum_{i=1}^{k}E{}_{w_{i}∼P_{n}\left(w\right)}\left[\log\sigma \left(-v{{}_{w_{i}}^{\prime }}^{⊤}v_{w_{I}}\right)\right].$$

where $v_{w}$ and $v_{w}^{{}^{\prime }}$ are the “input” and “output” vector representations of *w*, set the initial value by random initialization. Negative sampling *k* times per node, where σ(x)=1/(1+exp(−x)), $P_{n}\left(w\right)$ is noise distribution.

## 4. The Proposed Methed

*Graph embedding* is defined as end-to-end learning [34]—first random sampling, then representation learning. We focus on representation learning. In Section 4.1, we propose the Graph Skip-gram model, a graph embedding method for learning graph structural information, capturing local and global information among nodes during graph representation learning, and that can be extended to weighted graphs. In Section 4.2, we propose an algorithm for selectively fusing node features based on the similarity of node vector representations. In Section 4.3, we discuss the complexity of the algorithm.

### 4.1. Graph Skip-Gram Model

In networks (graphs) without node features, structural information on the graph plays a crucial role in unsupervised learning of node feature representations. Despite the many models proposed, the question remains how to effectively incorporate the structural information of the graph into graph embedding. In this section, we use our insights in combination with Skip-gram to construct a model more appropriate to graph embedding, which we call Graph Skip-gram.

**Figure 1:**
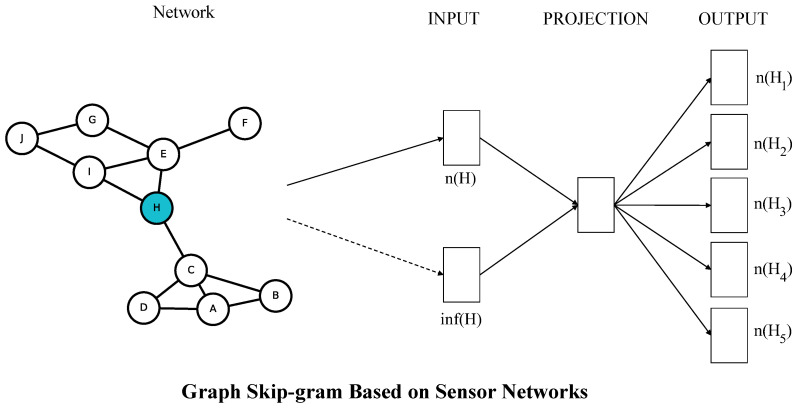
The framework of the Graph Skip-gram. The Graph Skip-gram predicts surrounding nodes given the current node. The graph structure information around node H is extracted on the graph as inf(H), in sensor networks is available by reading node information, inf(H), and node H as inputs to the model, and the output is the prediction of node H on the surrounding nodes H${}_{i}$.

The framework of Graph Skip-gram is shown in Figure 1. In a given graph G, we define the objective of the Graph Skip-gram, which maximizes the log-probability of observing a network neighborhood $N_{S}\left(u\right)$ for a node *u* conditioned on its feature representation, given by *f*:

(4) $$\max_{f}\sum_{u\in V}\log Pr(N_{s}\left(u\right)|f\left(u\right)).$$

In order to make the optimization problem tractable on the graph, we refer [19] to make two standard assumptions:

- Conditional independence. We factorize the likelihood by assuming that the likelihood of sampling to a neighborhood node is independent of sampling to any other neighborhood node, given the feature representation of the source:
  (5) $$Pr\left(N_{s}\left(u\right)|f\left(u\right)\right)=\prod_{n_{i}\in N_{s}\left(u\right)}Pr(n_{i}|f\left(u\right)).$$
- Symmetry in feature space. In the feature space, a source and neighborhood node have a symmetric influence on each other. Therefore, we model the conditional likelihood of each source-adjacent node pair as the dot product of the softmax unit and the graph structure information function:
  (6) $$Pr\left(n_{i}|f\left(u\right)\right)=\frac{\exp\left(f^{{}^{\prime }}\left(n_{i}\right)\cdot f\left(u\right)\right)\cdot W\left(n_{i},u\right)}{\sum_{v\in V}\exp(f^{{}^{\prime }}\left(v\right)\cdot f\left(u\right))\cdot {\left[W(v,u)\right]}^{L_{v}^{u}}}.$$
  The dot product of node features parametrizes the softmax unit. We define W(n,u) as the graph structure information function representing the closeness between nodes *n*, *u*:   
  (7) $$W(n,u)=w\cdot \frac{D_{u}}{D_{n}}+\left(1-w\right)\cdot {\left[\left(2-\frac{2\cdot P_{un}}{p}\right)\right]}^{a}.$$
  D (node degree matrix) and P (distance matrix between nodes) can both be obtained on the graph. For example, solve the D matrix by counting the concatenated edges of the nodes, and solve the P matrix using the Dijkstra algorithm. Through the distance information between nodes and the difference in nodes’ degree, the Graph Skip-gram can capture the local and global information of the graph.

**Figure 2:**
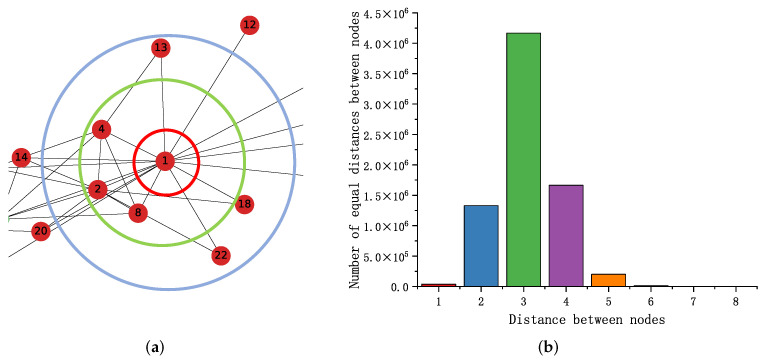
(**a**) Take part of the network in karate as an example, the nodes are distributed at different levels. (**b**) Distance distribution between PPI network nodes.

As shown in Figure 2a, nodes located at the center of the network (node 1) have more links relative to the edge nodes of the network (nodes 13, 18, 22, 12). We wish to capture this relationship through the difference in node degree and improve the quality of node embedding. Let $D_{u}$ and $D_{n}$ be the degrees of node *u* and node *n*. We usually define $1\leq D_{u}/D_{n}\leq 1.1$, increase the co-occurrence probability of nodes at different levels of the network and, thus, reduce the intra-class spacing. We define that the co-occurrence probability between two nodes increases with decreasing node spacing, and the value of *p* is determined by the distance distribution between nodes.

Setting of parameters *p*, *a*: The distribution of distances between nodes in the PPI [55] network is shown in Figure 2b. By observing the distribution of distances between nodes against the Gaussian distribution, setting p=7, make $P_{un}\leq 6$. When the distance distribution is unknown, predict it by random sampling. In general, setting a=1, but when the distribution of numerical terms in the *p* matrix is not uniform, adjusting *a* to alleviate the problem of concentrated distribution of distances between nodes; furthermore, we discuss the parameter *a* in the subsequent experimental section.

The *w* is a hyperparameter (0<w<1) that adjusts the weight between node degree and node distance. In addition, the Graph Skip-gram can be extended to a weighted graph, and the graph structure information is defined as follows:

(8) $$W(n,u)=w\cdot \frac{D_{u}}{D_{n}}+\left(1-w\right)\cdot {\left[\left(2-\frac{2\cdot P_{un}}{p}\right)\right]}^{a}+w^{{}^{\prime }}\cdot W_{un}.$$

The $w^{{}^{\prime }}$ is a hyperparameter used to adjust the weight proportion of edges in the weighted graph. $W_{un}$ denotes the weight of the edge connecting node *u* to node *n*.

Define $L_{v}^{u}$ to indicate whether the nodes are reachable:

(9) $$L_{v}^{u}=\left\{\begin{array}{c} 0\\ 1 \end{array}\begin{array}{c} \;P_{uv}=0\\ \;P_{uv}>0 \end{array}\right.\;.$$

If $P_{uv}=0$, node *u* is not reachable to node *n*, let $L_{v}^{u}=0$; if $P_{uv}>0$, node *u* is reachable to node *n*, let $L_{v}^{u}=1$; if node *u* is positively sampled (random walks) to node *n*, then $L_{n}^{u}=1$ (n≠u).

Optimizing Equation (6) using the negative sampling technique simplifies to:

(10) $$\log Pr\left(n|u\right)=\log A\left(f^{{}^{\prime }}\left(n\right),f\left(u\right)\right)+\sum_{i=1}^{k}E{}_{n_{i}∼P_{n}\left(u\right)}\left[\log A(-f^{{}^{\prime }}\left(n_{i}\right),f\left(u\right))\right].$$

(11) $$A\left(f\left(n\right),f\left(u\right)\right)=\sigma \left(f{\left(n\right)}^{⊤}f\left(u\right)\right)\cdot {\left[W(n,u)\right]}^{L_{n}^{u}}.$$

In addition, restrict the range of values of A(a,b):

(12) $$A\left(a,b\right)=\left\{\begin{array}{cc} 1 & A(a,b)>1\\ A(a,b) & -1\leq A(a,b)\leq 1\\ -1 & A(a,b)<-1 \end{array}\right..$$

### 4.2. Exploring Two Sets of Node Vector Representations

In the graph embedding approach based on the Skip-gram model, training yields two sets of node vector representations for each node-input and output embedding. Unlike previous work that used one set of embeddings or combined two sets of embeddings, we consider fusing the input and output embeddings of part nodes (Fioepn). Inspired by the similarity of word vectors, we calculate the cosine similarity between the two sets of embeddings for each node, then fuse the embeddings of the nodes with the lowest similarity. In Section 5.2, we introduce this algorithm in a practical application.

The pseudocode for Fioepn is given in Algorithm 1. First, calculate the similarity scores of the input embedding and output embedding of each node (lines 1–3); then sort the similarity scores to obtain the sorted similarity scores (line 4, the scores do not change before and after sorting); then obtain the minimum similarity scores according to the node feature update rate (sim, line 5); perform feature fusion on partial nodes in lines 6–12; lastly, return the optimized input embedding (line 13).


**Algorithm 1** Embedding Fusion $\left(f,f^{{}^{\prime }},p\right)$.**Input:** input embedding *f*, output embedding $f^{{}^{\prime }}$, Fioepn ratio *p*;**Output:** optimal input embedding $f^{*}$
 1:**for** v∈V **do** 2:    $score_{v}$ ← $f_{v}•f_{v}^{{}^{\prime }}$; 3:
**end for**
 4:sortscore←Sort(score);                ▹ No change to the score 5:$sim\leftarrow sortscore_{p*|V|}$; 6:**for** v∈V **do** 7:    **if** $score_{v}<sim$ **then** 8:        $f_{v}^{*}\leftarrow \left(f_{v}+f_{v}^{{}^{\prime }}\right)/2$; 9:    **else** 10:        $f_{v}^{*}\leftarrow f_{v}$; 11:    **end if** 12:
**end for**
 13:**return** $f^{*}$;



### 4.3. Complexity Analysis

Solving the implicit information matrix on the graph, the time complexity of the **D** (degree Matrix) is O(|V|); we use the Dijkstra algorithm to solve the matrix **P**, and the time complexity of solving the matrix P is O(|E|log(|V|)), the implementation of the Dijkstra algorithm is based on a heap implementation of the priority queue data structure.

Similar to the Skip-gram model, the Graph Skip-gram can use negative sampling and stochastic gradient descent (SGD) for optimization. The time complexity of Equation (10) is O(k), *k* is the number of negative samples. The time complexity of Fioepn is O(|V|). The time complexity of Graph Skip-gram is malx(O(|E|log|V|),O(mdk|V|)), *m* is the window size of the Graph Skip-gram model, *d* is the dimension of embedding.

## 5. Application of the Method

In this section, we first explain the principle of the Skip-gram model for learning node feature representation through a case study; and then introduce our proposed algorithm analytically through a small network. In sensor networks, the distribution of sensor nodes can be quickly perceived by visualizing the target network with the Graph Skip-gram model.

### 5.1. Understanding the Node Embedding Process Intuitively

In this section, we use DeepWalk to learn the node features of the Karate network. We refer to the word2vec and DeepWalk code implementations given in [2,56] to give a simplified graph embedding pseudo-code implementation, Algorithms 2 and 3. Referring to the paper [18], we set embedding dimension d=2, windows size m=5, walks per node r=40, walk length t=40, and negative sampling k=1 for training.

**Figure 3:**
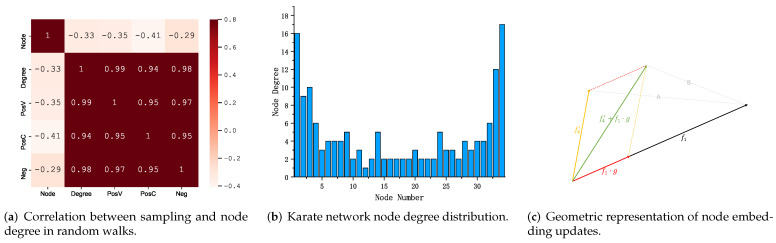
Sampling analysis: (**a**,**b**); visual nodes represent the update process: (**c**).

**Figure 4:**
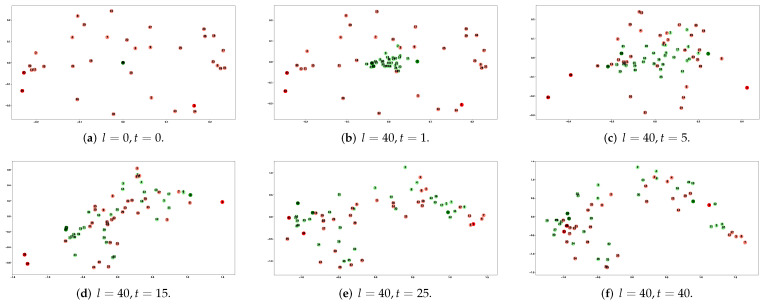
Visualization of node distribution at different training phases.

We counted the number of times each node used as a central word (PosV), surrounding word (PosC), and negative sample word (Neg) during the whole training process. We use the statistical sampling information of each node with the node number (Node) and node degree (Degree) to create a heat map, as shown in Figure 3a; Figure 3b shows the node degree distribution in the karate network. The heat map shows that PosV, PosC, Neg, and Degree are highly correlated, demonstrating sampling frequency of the node is related to the node degree, as introduced in the Section 1, nodes containing more connected edges are critical in learning node feature representation. Figure 4 shows the node features learned at different stages. For example, input embedding in red, output embedding in green, and we highlight the example nodes by deepening the node color.

Completing the above preparations, let us next explore how Skip-gram works.

In the initial stage of learning, as shown in line 1 of Algorithm 2, it randomly initializes the input embedding parameters while setting all parameters of the output embeddings to zero; as shown in Figure 4a, the input embedding is randomly distributed on the coordinate axes, input embedding overlaps centrally at the coordinate origin. Since the final learned input and output embeddings are similar, initializing the input embeddings by randomization and the output embedding parameters to zero helps to accelerate the training.

We use node 1 as an example node for subsequent study and discussion. When node 1 is the central word (*u*), used as the sampled node to sample its surrounding nodes (*n*, node 4, Algorithm 2, line 6). Then invoke Algorithm 3 to update the node feature representation. In Algorithm 3, first, calculate the similarity between the node *u*’s input embedding and the node *n*’s output embedding (line 1). Then calculate the gradient according to its positive/negative sample and learning rate (line 2); take positive sampling as an example (label = 1; *u* = 1, *n* = 4), the gradient increases as the similarity decreases, gradient value affects the calculated error term. Then update the cumulative error term (line 3). In line 4, update the output embedding. At last, return the cumulative error term (line 5).


**Algorithm 2** Learn Node Feature Repreaution (G,m,d,t,l,k).**Input:** graph G(V,E), window size *m*, embedding size *d*, walk length *t*, walks per vertex *r*,  negative sampling *k*;**Output:** Output *f*;
 1:Initialization: Random init *f*, init $f^{{}^{\prime }}=0$, TotalSteps = |V|*r, Sampling(lable); 2:**while** step < TotalSteps **do** 3:    **for** i = 1 to *t* **do** 4:        **for** j = 1 to RandFunuction(*m*) **do** 5:           neule=0;               ▹$neule\in R^{d}$ Error accumulation term 6:           (*n*, *u*) ← Sampling(1)                    ▹ Positive sampling 7:           neule← UpdateFeatures($f_{n}^{{}^{\prime }},f_{u},1$); 8:           NegSample(neg, u) ← Sampling(0)            ▹ Negative sampling 9:           **for** (neg, u) ∈ NegSample(neg, u) **do** 10:               neule←neule + UpdateFeatures($f_{neg}^{{}^{\prime }},f_{u},0$); 11:           **end for** 12:           $f_{u}\leftarrow f_{u}+neule$; 13:        **end for** 14:    **end for** 15:    step++; 16:
**end while**
 17:**Save** *f*;




**Algorithm 3** UpdateFeatures ($f_{n},f_{u},lable$).**Input:** output embedding $f_{n}$, input embedding $f_{u}$, Lable: PosSam(1), NegSam(0);**Output:** Output *f*;
 1:score ← $f_{n}•f_{u}$; 2:g←(lable− sigmod(score))·α; 3:catch← $catch+f_{ni}\cdot g$;                   ▹$catch\in R^{d}$ 4:$f_{n}\leftarrow f_{n}+f_{u}\cdot g$; 5:**return** catch;



Viewing the node feature update process from the perspective of geometric representation, which can be intuitively understood as positively sampled nodes approaching the central node (input embedding) along the direction of the approximate central node. As shown in Figure 3c, the output embedding corresponding to node 4 moves along the direction approximating the input embedding of node 1; therefore, the similarity between nodes increases (distance: B < A).

After completing the positive sampling node feature update, perform negative sampling (Algorithm 2, line 8), updating the node features based on the nodes obtained by negative sampling in the same way as the above analysis. In the final stage of completing a round of node sampling updates (Algorithm 2, line 12), the input embedding of the central node (node 1) is updated based on the error term; in the process of calculating the error term, the output embedding of the surrounding nodes and the negative sampling nodes are updated (negative sampling updated along the opposite direction).

**Figure 5:**
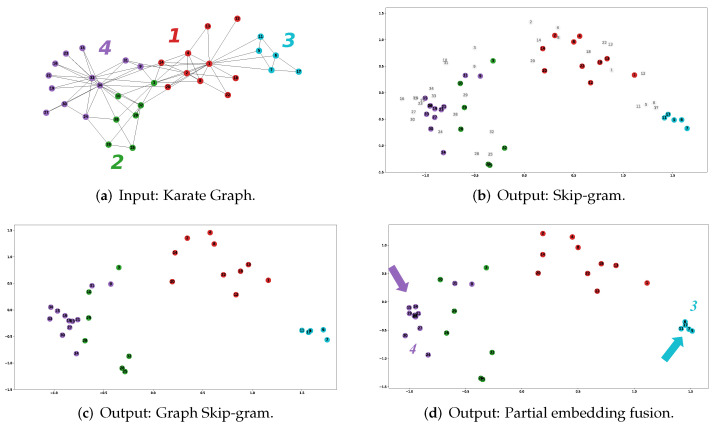
Different methods node embedding visualization.

Figure 4c shows the node representation after five rounds of training; it is clear that the hub nodes (node 1, node 33, node 34) are further away from the origin relative to their initial positions (both input and output embeddings) because the sampling frequency is related to the node degree; at this stage, the output embedding changes more obviously, they move towards their respective input embedding and away from the origin. Figure 4d shows the node features obtained after 15 training rounds, the hub nodes reach the most edge position of the network. In the next stage, the more significant change in the training node features is the aggregation of the network edge nodes towards the central node (Figure 4e), eventually learning a high-quality node feature representation (Figure 4f or Figure 5b).

Above, we discussed the case of two-dimensional graph embedding. Likewise, it can be extended to the case of three-dimensional or even higher-dimensional.

In brief, the essence of the above process is as follows. The sampled node obtains its surrounding nodes by random sampling, then calculates the gradient based on the similarity of the vector representation between the nodes to update the sampled node’s input embedding and the surrounding node’s output embedding. Analyzing this phenomenon at a geometric level is shown in Figure 3c, which makes the output embeddings of the surrounding nodes closer to the input embeddings of the sampled nodes (distance: B < A); the opposite is true for the node feature update process with negative sampling. Then, obtain two vector representations of each node by multiple sampling updates.

### 5.2. Graph Skip-Gram and Fioepn

As shown in Figure 5a, we visualized the Karate network using force-directed layouts, classifying the nodes into four classes using modularity-based clustering [57]. We numbered the nodes according to categories for subsequent studies.

We use the DeepWalk method based on Skip-gram (Figure 5b) and Graph Skip-gram (Figure 5c) to learn the node feature representation of the Karate network, we use the same parameter settings as Section 5.1, in the Graph Skip-gram model, setting the parameter w=0.8, p=5, a=1. We use Calinski–Harabasz (CH) [58] and within-group cluster sum of squares (WGSS) [58] to evaluate the clustering performance of node features learned from different models.

As shown in Table 2 (first four lines), compared to the Skip-gram, the Graph Skip-gram has a 5% improvement in CH score, and the WGSS scores indicate the effect of intra-class clustering achieves performance gains in three of the four classes. The light-colored points in Figure 5b are input embeddings, and the dark-colored points are output embeddings. In Figure 5d, we show the node feature representation after updating 50% of the nodes using the Fioepn algorithm; obviously, the third and fourth-class clustering effect is improved. Table 2 (bottom six lines) compares the WGSS scores between the different categories; using the Fioepn algorithm, the WGSS(3) score improves by 69%.

The above node visualization and experimental analysis of the clustering effect clarify the idea of Graph Skip-gram and Fioepn: more effective use of graph structure information and input and output embeddings. It also demonstrates the effectiveness of Graph Skip-gram and Fioepn.

## 6. Experiments

### 6.1. Experimental Setup

#### 6.1.1. Environment

We completed all experiments on a desktop PC with a hexa-core Intel i5 3.00 GHz processor and 32GB of RAM. The operating system is Ubuntu 16.04 and Windows 10. Training of the DeepWalk, node2vec, Walklets, and TADW was run in Ubuntu 16.04, and the training of other models and related experimental evaluations was run in Windows 10.

#### 6.1.2. Datasets

We use four datasets to evaluate the performance of Graph Skip-gram and Fioepn, and Table 3 gives the relevant statistics about the datasets.

- BlogCatalog [59]: this dataset is a social network that consists of the blogger and their social connections (e.g., friends).
- Wikipedia [60]: this is a co-occurrence network of words.
- PPI [55]: this is a subgraph of the protein–protein interaction network for Homo Sapiens.
- Flickr [61]: this is a social network where nodes represent users and edges correspond to user friendships.

Note that, like the datasets used for the experiments in paper [18,19], the above four datasets do not contain node attribute information, and some of the nodes have multiple labels. These conditions are unfavorable for training neural network-based graph embedding methods, and we give the solution in the following method introduction.

#### 6.1.3. Method

Eight graph representations learning methods are used as baselines in the experiments.

- TADW [30]: a matrix factorization-based method that uses semantic information to improve the quality of node embedding.
- GCN [37]: a convolutional neural network that applies directly on graphs and uses their structural information.
- GAT [38]: to alleviate the two drawbacks of GCNs, proposing to incorporate attention mechanisms into spatial GCNs to provide differentiated weights for neighborhoods.
- GraphSAGE [48]: it proposes a generalized induction framework that uses node feature information to generate node embeddings on the graph.
- ACMP [62]: it introduces the Allen–Cahn message passing mechanism in GNN, a deep GNN model that avoids the oversmoothing problem of GNN.
- DeepWalk [18]: it is based on Skip-gram and uses random walks to generate node pairs for training node feature representation.
- Node2vec [19]: based on DeepWalk, it proposes a biased random walks strategy that combines the search strategies of BFS and DFS properties.
- Walklets [20]: it optimizes DeepWalk’s sampling strategy on large graphs, capturing multiple hierarchical relationships of nodes in the network.

We refer to the code implementation of DeepWalk and node2vec in [56]. The source code for the other six approaches can be found in the relevant Github projects.

Since the dataset used in this experiment has no node features, the node feature matrix used in the TADW, GCN, GAT, GraphSAGE, and ACMP uses the degree matrix instead; among the four methods, only the TADW method performs training in an unsupervised manner. Following the principle of randomness, we extract single-label nodes from the above four datasets and use them for training the GCN, GAT, GraphSAGE, and ACMP in a supervised manner. The training, validation, and test sets are divided 6:2:2, respectively, and save the best performance results for subsequent performance evaluation. The node vectors obtained from TADW, GCN, GAT, GraphSAGE, and ACMP are evaluated for multi-label classification and link prediction performance using the same evaluation scheme as DeepWalk, node2vec, and Walklets.

We use Graph Skip-gram and Skip-gram to learn node feature representations in standard unsupervised learning tasks. For all methods based on Graph Skip-gram and Skip-gram, we refer to [18,19,20], set the embedding dimension d=128, default learning rate as 0.025. In the optimization phase, all methods are optimized using negative sampling and SGD, learning the feature representation with four threads. For DeepWalk and node2vec, we refer to the experimental setup in [19], set windows size m=10, walks per node r=80, walk length t=10, negative sampling k=5; *p* and *q* in node2vec are searched over $\left\{0.25,\;0.50,\;1,\;2,\;4\right\}$. Walklets focus on capturing information at different scales in the graph; we refer to the experimental setup from [20], set walks per node r=1000, walk length t=11, negative sampling k=5, $k^{{}^{\prime }}$ (skip factor) in $A^{k^{{}^{\prime }}}$ are searched over $\left\{1,\;2,\;3\right\}$.

### 6.2. Multi-Label Classification

Section 6.2.1 evaluates Graph Skip-gram’s performance by adjusting the walk length *t* and comparing it with Skip-gram. Section 6.2.2 analyzes the performance of Graph Skip-gram by comparing multiple methods.

We use the same experimental procedure listed in [18] to evaluate the performance of the methods. We use different proportions of randomly selected nodes for training the linear classifier and use the rest for testing. We repeated the experiment 10 times, randomly sampling the training and test nodes each time and reporting the average Micro-F1 for all methods. We do not include results for other evaluation metrics, such as precision and recall, because they all follow the same trend. We use BlogCatalog, Wikipedia, PPI, and Flickr datasets for the multi-label classification task. Referring to the performance in [18], we treat all the datasets as undirected graphs.

The settings of the different methods in the experimental results are as follows. For example, DeepWalk uses Skip-gram as the base model for feature representation learning; DeepWalk* uses Graph Skip-gram as the base model for feature representation learning; DeepWalk** uses Graph Skip-gram as the base model for feature representation learning, using Fioepn optimized input embedding. Set all datasets input–output embedding vector fusion ratio p=10%.

#### 6.2.1. Performance Comparison between Graph Skip-Gram and Skip-Gram

**Figure 6:**
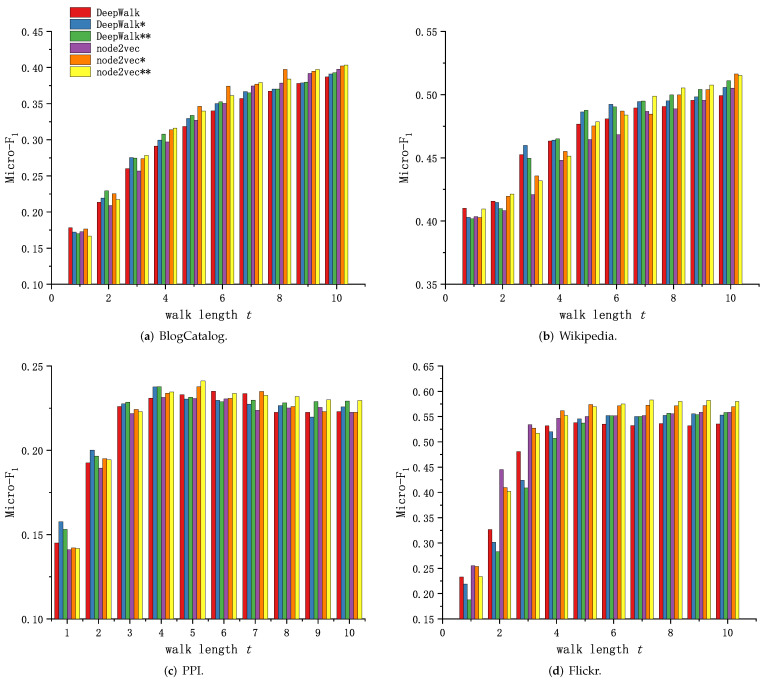
Graph Skip-gram vs. Skip-gram based on DeepWalk and node2vec.

**Figure 7:**
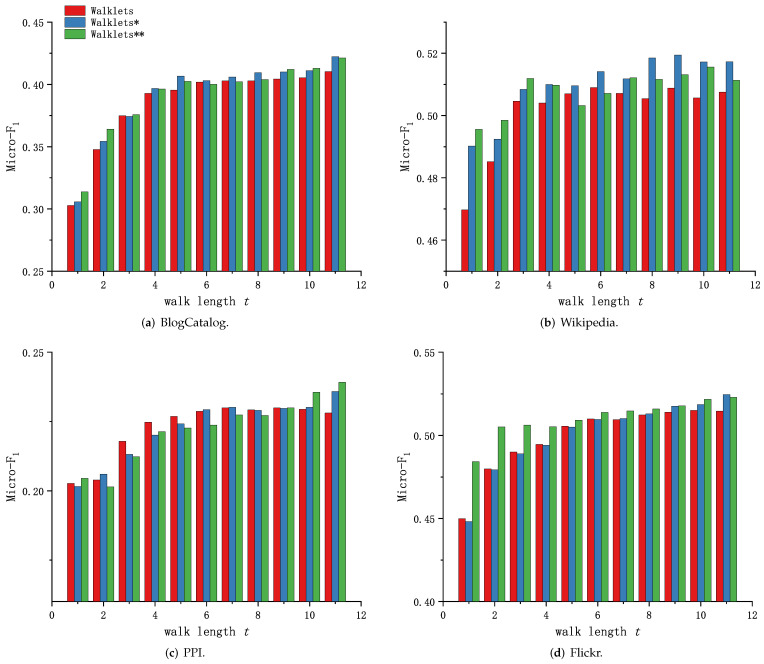
Graph Skip-gram vs. Skip-gram based on Walklets.

Because of the different training methods of DeepWalk, node2vec and Walklets, and the readability of the experimental results, the experimental results in this subsection are presented and analyzed in two groups. Figure 6 and Figure 7 shows the performance comparison of Graph Skip-gram and Skip-gram at different walk lengths *t*.

As shown in Figure 6a and Figure 7a, the method based on Graph Skip-gram shows performance advantages as the walk length *t* increases (t>2), consistently outperforming the method based on Skip-gram during the training process, demonstrating the Graph Skip-gram model captures the graph structure information and improves the quality of node feature representation. As shown in Figure 6b and Figure 7b, the performance of the methods based on Graph Skip-gram and Fiopen steadily improves with increasing *t* and consistently outperforms the methods based on Skip-gram, demonstrating that fusing some of the node features helps to obtain higher-quality node features. As shown in Figure 6c, as the walk length *t* increases, the performance of all of the methods rise first and then fall, but the methods based on Graph Skip-gram and Fiopen consistently outperform those based on Skip-gram.

Figure 6d and Figure 7c show similar performance trends. The methods based on Graph Skip-gram have weak performance when the walk length *t* is small. This is because Graph Skip-gram captures the graph structure properties when the number of training rounds (*t*) is small, which makes the node vector update less frequently (Figure 3c) and the same class of nodes is poorly aggregated; as the walk length *t* increases, the ability of Graph Skip-gram to capture the characteristics of graph structure begins to manifest, making the node vector representation more differentiated across categories, gained an advantage at walk length t≥6 (Figure 6d) and staying ahead.

We observe in each dataset that node2vec has improved performance compared to DeepWalk when using different search strategies (node2vec: $p,\;q\in \left\{0.25,\;0.50,\;1,\;2,\;4\right\}$; DeepWalk: p=1, q=1). Similarly, experiments demonstrate that our proposed Graph Skip-gram compensates for the defects of random walks, and DeepWalk* using the Graph Skip-gram achieves performance not inferior to node2vec. This conclusion also holds for the Walklets (Figure 7) method.

#### 6.2.2. Graph Skip-Gram versus Multiple Methods

In this section, we explore the performance of Graph Skip-gram by comparing multiple methods. We summarize the results of the experiment in Table 4. We increase the training rate of each network from 10% to 90%. The hyperparameter settings for Graph Skip-gram in each dataset are indicated in Table 4.

**Figure 8:**
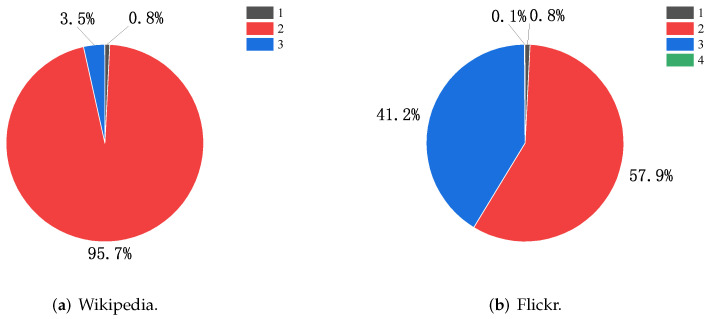
Distribution of numerical entries in the **P** matrix for the Wikipedia and Flickr datasets.

Figure 8 shows the distribution of **P** matrix values in the Wikipedia and Flickr datasets. In the Wikipedia dataset (Figure 8a), the distribution of values in the **P** matrix is concentrated, with $P_{un}=2$ accounting for 95.7% and $P_{un}=3$ for 3.5%; we set a=0.5 to adjust the distance information function between nodes so that Graph Skip-gram can better capture the structural information of the graph. A similar situation occurs in the Flickr dataset (Figure 8b), where we adjust a=0.1.

From the results shown in Table 4, it is clear that the combination of Graph Skip-gram and Fioepn outperforms Skip-gram and TADW on each dataset. GCN, GAT, GraphSAGE, and ACMP are trained in a supervised manner, and only GCN achieves a performance advantage in the PPI dataset where Graph Skip-gram performs poorly, but Graph Skip-gram is trained in an unsupervised manner. The performance of TADW, GCN, GAT, GraphSAGE, and ACMP failed to achieve satisfactory prediction performance in networks without node features, and the performance gap between them and Graph Skip-gram methods is large.

The results of evaluation experiments on the BlogCatalog dataset show that GraphSAGE—the best performing of the five methods (TADW, GCN, GAT, GraphSAGE, and ACMP)—has a 17% performance gap compared to Walklets* (training rate: 0.9).

In the Wikipedia dataset, the performance of the above five methods is similar, but lower performance than Walklets*. In the Flickr dataset, the performance difference between GCN and node2vec** is as high as 18.44% (training rate: 0.9). This phenomenon is broken by GCN in the PPI dataset, where the Graph Skip-gram model performs poorly, but the performance of the other three methods is still inferior to Graph Skip-gram. Obviously, it is difficult for models based on graph neural networks to gain an advantage in networks without node attributes. In contrast, Graph Skip-gram model do not use node attributes, and training is unsupervised. Our proposed Graph Skip-gram and Fioepn show surprising results on the Flickr dataset with DeepWalk and node2vec algorithms, achieving 3.54% (DeepWalk**) and 2.97% (node2vec**) improvement at a training rate of 0.1, and ahead of GCN by 14.05% and 16.31%, respectively. On the Wikipedia dataset, the Graph Skip-gram model-based method (node2vec*, Walklets*) improves by 1.12% and 0.98% compared to node2vec and Walklets at a training rate of 0.9, and significantly better than the above five methods. These experimental results demonstrate that Graph Skip-gram learns graph structure information and is more suitable for graph embedding than Skip-gram; the Fioepn algorithm can improve the quality of node embedding.

### 6.3. Parameter Sensitivity

We analyze the sensitivity of parameters w,p, and *a* on the Flickr and Wikipedia datasets (training rates are 30% and 90%, respectively), using node2vec as the base method. We do not show sensitivity analysis of parameters based on other models and datasets, such as DeepWalk* and DeepWalk** on different datasets, because they all follow similar trends.

**Figure 9:**
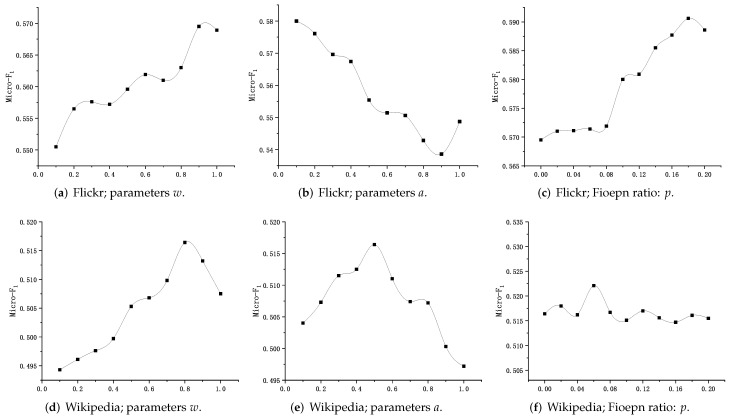
Impact of hyperparameters and Fioepn ratio on the performance of the proposed method.

Figure 9 shows the sensitivity of the parameters based on the Flickr and Wikipedia datasets. Figure 9a,d show the effect of the parameter *w* on the Micro-F${}_{1}$ score, and both datasets’ optimal *w* values exist. Referring to Equation (7) demonstrates that adjusting the co-occurrence probability of nodes at different levels can improve the quality of node embedding. The problem of uneven distribution of **P** matrix data items in Figure 8a can be alleviated by adjusting the parameter *a*, as shown in Figure 9e, obtaining the best performance when a=0.5. Figure 9f shows the performance obtained by adjusting the node vector fusion rate in the Wikipedia dataset fluctuates as the fusion rate changes. Figure 9c shows how the value of *p* affects the performance; a 2% performance improvement can be gained by properly setting the parameter *p*, demonstrating the effectiveness of the Fioepn algorithm in optimizing node embedding.

Through the experimental analysis, we can see that the parameter settings have an impact on the performance of our model, and proper setting of parameters helps to gain better performance.

### 6.4. Link Prediction

Determining if a link exists between two network nodes is known as link prediction. We used the same experimental procedures listed in [19] to evaluate the link prediction performance of the different methods. We randomly remove some edges from the network and ensure the remaining network is still connected. We sample positive and negative training samples in the network according to the following rules. We randomly sample pairs of nodes with connected edges in the network as positive samples; we randomly generate pairs of nodes and ensure that they have no connected edges as negative samples.

We test the performance of the Graph Skip-gram against the link prediction task using the Flickr, Wikipedia, and PPI datasets introduced in the Section 6.1.2. We train a logistic regression classifier on the edgewise features obtained with the method shown in Table 5. For example, for a pair of nodes *u* and vs., the average operator generates a vector as the link between that pair of nodes.

### 6.5. Experimental Results

We summarize the results for link prediction in Table 6. We use the node feature representations generated in the previous section for this task, which fully demonstrates the adaptability and scalability of our method.

As shown in Table 6, TADW, GCN, GAT, and GraphSAGE have no advantage in the link prediction task if the network is without node attributes. For example, in the Wikipedia dataset, GAT has the best performance among the four methods, with an accuracy of 83.5% under the Weighted L1 operator, but still has a 13% performance gap compared to Walklets**.

The ACMP approach outperforms the previous four approaches on the Flickr dataset, achieving optimal performance for link prediction under all four operations, outperforming the method based on Graph Skip-gram in link prediction under the Hadamard operation; however, there is a 15.6% and 15.8% performance gap with Walklets** under Weighted L1 and Weighted L2 operator.

In the Wikipedia dataset, node2vec* gains 3.1% performance gains over node2vec under the Weighted L1 operator; node2vec* gains 3.0% performance gains over node2vec under the Weighted L2 operator. Similar performance gains are observed on the PPI and Flickr datasets, demonstrating that Graph Skip-gram learns graph structure information.

What is interesting is the performance of the Fioepn algorithm. For example, in the PPI dataset, DeepWalk** gains 5.4% and 8.1% performance gains over DeepWalk* and DeepWalk under the Weighted L1 operator; node2vec** gains 6.9% and 9% performance gains over node2vec* and node2vec under the Weighted L2 operator. There are similar results in the Wikipedia and Flickr datasets. In conclusion, our proposed Graph Skip-gram and Fioepn outperform Skip-gram on the Weighted L1 and Weighted L2 operators. This demonstrates that the fusion of partial node features helps to improve the link prediction accuracy.

## 7. Conclusions

Through an in-depth analysis of the differences between word embeddings and graph embeddings, we propose Graph Skip-gram, capturing local and global information of graphs through graph structure information functions, making Skip-gram more appropriate for graph embedding; inspired by word vector similarity, we devise a new input–output fusion embedding mechanism, Fioepn, which determines whether to use feature representation fusion by calculating the cosine similarity between the input–output embeddings. Through the case study, we give an intuitive understanding of graph embedding, which facilitates the understanding of the nature of graph embedding; we give a visualization of the node features learned by Graph Skip-gram and Fioepn, it is advantageous for quickly perceiving the distribution of nodes in a sensor network; by evaluating the effect of clustering, the ideas of the algorithm are further clarified.

Experimental results on multi-label classification and link prediction with multiple models and datasets demonstrate that Graph Skip-gram, which learns node features unsupervised, has significant advantages in networks without node attributes, and our proposed Graph Skip-gram and Fioepn are more suitable for graph embedding compared to Skip-gram. Our method uses the position information between nodes during training, which will increase the training time; we preprocess the node information needed for model training before model training, avoiding the repeated solving of node information and improving the training efficiency.

Our future work will focus on two directions: (1) Graph Skip-gram models are trained in an unsupervised manner, whereas methods based on graph neural networks often require partially labeled data for training; whether it is possible to devise an approach to establish a link between the both so that methods based on graph neural networks can be trained in an unsupervised manner. (2) Graph neural network models are usually trained using the node feature matrix for training, whether better model performance can be obtained using the inter-node distance matrix.

## Figures and Tables

**Table 1:** Notations and abbreviations.

Notation	Meaning
**In Skip-gram**	Notions in the Skip-gram model.
T	Number of training word sequences.
$\;w_{i}$	The ith training word sequence.
c	The size of the training context.
$\;v_{w},v_{w}^{{}^{\prime }}$	The “input” and “output” vector representation of word *w*.
W	The number of words in the vocabulary.
k	Negative sampling frequency.
$P_{n}\left(w\right)$	Noise distribution.
**In Graph Skip-gram**	Notions in the Graph Skip-gram model.
$\;f\left(u\right),f^{{}^{\prime }}\left(u\right)$	The “input” and “output” vector representation of node *u*.
V	The members of the graph.
$\;N_{s}\left(u\right)$	Network neighborhood of node *u*.
$\;L_{v}^{u}$	Whether node *u* can reach node *n*.
$\;w,w^{{}^{\prime }}$	Hyperparameter.
D	Node degree matrix.
P	Distance matrix between nodes.
a	Parameters adjusted according to **P** matrix.
W	Weighted graph weight matrix
k	Negative sampling frequency.
$P_{n}\left(u\right)$	Noise distribution.
**Abbreviation**	
NLP	Natural language processing.
CH	Calinski–Harabasz.
WGSS	Within-group cluster sum of squares.
Fioepn	Fusing the input and output embeddings of part nodes.

**Table 2:** The clustering performance of Graph Skip-gram model and Fioepn algorithm.

Basic Model	CH	WGSS(1)	WGSS(2)	WGSS(3)	WGSS(4)
Skip-Gram	45.116	**1.652**	3.699	0.073	1.999
Graph Skip-Gram	**47.409**	2.310	**3.597**	**0.069**	**1.757**
Gain over Skip-Gram[%]	**5.082**	—	**2.758**	**5.48**	**12.10**
**Embedding**	**CH**	**WGSS(1)**	**WGSS(2)**	**WGSS(3)**	**WGSS(4)**
*f* (Input Embedding)	**45.116**	**1.652**	**3.699**	0.073	1.999
$f^{{}^{\prime }}$ (Output Embedding)	37.840	2.170	5.026	0.056	2.115
$f^{*}$ (Fioepn)	42.807	1.896	3.970	**0.022**	**1.681**
Gain over *f* [%]	—	—	—	**69.863**	**18.917**
Gain over $f^{{}^{\prime }}$ [%]	**13.126**	**12.627**	**21.011**	**60.714**	**20.520**

**Table 3:** Statistics of the datasets.

Dataset	BlogCatalog	Wikipedia	PPI	Flickr
#nodes	10,312	4777	3890	7575
#edges	333,983	184,812	76,584	239,738
#labels	39	40	50	9

**Table 4:** Multi-label classification results in terms of Micro-F${}_{1}$ (%).

Method	BlogCatalog w=0.6, p=5, a=1						Wikipedia w=0.8, p=4, a=0.5				
**Training Rate**	**0.1**	**0.3**	**0.5**	**0.7**	**0.9**		**0.1**	**0.3**	**0.5**	**0.7**	**0.9**
TADW	16.17	16.22	16.77	16.95	17.13		40.70	40.97	41.25	41.40	41.17
GCN	21.68	22.85	23.31	23.54	23.61		41.15	41.48	41.67	41.72	41.90
GAT	16.68	16.92	17.06	17.27	17.70		40.79	40.91	41.01	41.15	41.25
GraphSAGE	22.54	23.56	23.82	23.94	24.55		40.98	41.38	41.43	41.60	41.90
ACMP	19.13	21.18	22.17	22.68	22.73		41.11	41.49	41.54	41.72	41.98
DeepWalk	34.44	36.72	37.76	38.22	38.71		44.85	47.61	48.70	49.28	49.92
DeepWalk*	**34.73**	**37.31**	38.21	**38.98**	39.11		**46.03**	48.31	49.36	49.92	50.56
DeepWalk**	34.62	37.01	**38.27**	38.77	**39.27**		45.9	**48.65**	**49.71**	**50.37**	***51.10***
node2vec	35.18	37.86	38.82	39.45	39.78		45.54	48.10	49.12	49.53	50.52
node2vec*	**35.39**	**37.98**	39.1	**39.93**	40.23		46.37	49.20	**50.31**	**51.20**	51.64
node2vec**	35.22	37.84	**39.11**	39.62	**40.34**		**46.64**	**49.31**	50.26	50.58	51.51
Walklets	36.90	39.38	40.12	40.48	41.03		42.48	47.13	49.09	50.22	50.75
Walklets*	**37.29**	39.32	40.39	**40.94**	42.23		**43.58**	**47.92**	**49.59**	**50.65**	51.73
Walklets**	37.22	**39.49**	**40.49**	40.89	42.13		43.11	47.76	49.16	50.24	51.13
**Method**	**PPI** w=0.2, p=7, a=1						**Flickr** w=0.9, p=5, a=0.1				
**Training Rate**	**0.1**	**0.3**	**0.5**	**0.7**	**0.9**		**0.1**	**0.3**	**0.5**	**0.7**	**0.9**
TADW	6.51	6.74	6.48	6.73	7.93		11.28	11.31	11.35	11.41	11.44
GCN	**23.38**	**27.29**	**28.28**	**29.10**	**29.48**		38.71	40.10	40.84	41.30	42.06
GAT	7.03	7.88	9.61	10.89	12.05		17.29	26.19	29.21	30.51	32.60
GraphSAGE	12.66	16.58	18.24	18.76	19.12		11.08	11.14	11.16	11.31	11.43
ACMP	8.19	9.89	10.25	10.34	10.61		12.80	13.19	13.31	13.30	13.56
DeepWalk	15.88	19.34	20.64	**21.56**	22.30		49.22	53.55	54.67	55.21	56.14
DeepWalk*	15.90	19.29	20.55	21.32	22.58		51.75	55.33	56.23	57.02	57.12
DeepWalk**	**16.29**	**19.42**	**20.75**	21.52	**22.92**		52.76	**55.80**	**57.15**	**57.42**	**57.52**
node2vec	16.28	19.29	20.41	21.60	22.26		52.05	55.84	57.13	58.6	59.01
node2vec*	**16.44**	19.33	**20.57**	**21.64**	22.26		53.46	56.95	57.42	59.22	60.16
node2vec**	16.05	**19.43**	20.55	21.44	**22.95**		55.02	**58.00**	**58.58**	**60.10**	60.50
Walklets	17.15	20.04	21.66	22.34	22.81		45.75	51.46	53.64	54.60	55.11
Walklets*	**17.25**	**20.39**	**21.89**	22.72	23.59		**46.27**	**52.46**	54.47	55.57	56.33
Walklets**	17.20	20.20	21.81	**22.96**	23.92		45.91	52.29	**54.55**	**56.13**	**56.65**

**Table 5:** Vector operators used for link-prediction task for each u, v∈V.

Operator	Result
Average	f(u)+f(v)f(u)+f(v)22
Hadamard	$\left[f_{1}\left(u\right)*f_{1}\left(v\right),\ldots ,f_{d}\left(u\right)*f_{d}\left(v\right)\right]$
Weighted L1	$\left[\left|f_{1}\left(u\right)-f_{1}\left(v\right)\right|,\ldots ,\left|f_{d}\left(u\right)-f_{d}\left(v\right)\right|\right]$
Weighted L1	$\left[{\left(f_{1}\left(u\right)-f_{1}\left(v\right)\right)}^{2},\ldots ,{\left(f_{d}\left(u\right)-f_{d}\left(v\right)\right)}^{2}\right]$

**Table 6:** Link prediction results (%).

Method	Wikipedia				PPI				Flickr			
	Ave	Had	L1	L2	Ave	Had	L1	L2	Ave	Had	L1	L2
TADW	36.0	35.9	64.8	54.8	47.4	35.3	58.7	62.7	35.8	36.1	60.1	60.9
GCN	64.0	80.3	69.3	70.0	35.2	71.5	80.1	77.8	36.0	72.3	65.9	63.6
GAT	64.0	84.6	83.5	78.3	35.3	67.9	78.8	58.1	36.0	62.1	67.0	57.5
GraphSAGE	41.1	68.5	56.6	55.6	43.9	67.2	66.1	63.6	36.0	36.1	50.1	36.0
ACMP	36.1	70.4	70.5	64.0	64.2	64.0	52.1	51.6	**64.0**	**85.5**	**78.6**	**78.0**
DeepWalk	**65.1**	**85.1**	71.4	72.9	56.3	**86.9**	74.1	74.7	**64.0**	**87.9**	73.8	74.9
DeepWalk*	64.5	82.1	**76.1**	**77.7**	**65.0**	84.9	76.8	77.2	53.1	83.7	75.7	76.7
DeepWalk**	64.8	82.2	73.9	75.5	64.8	85.6	**82.2**	**82.4**	59.7	83.9	**75.7**	**76.8**
node2vec	64.4	**87.0**	75.0	76.7	51.4	**85.7**	72.6	72.7	64.0	**87.3**	73.1	74.1
node2vec*	64.5	81.5	**78.1**	**79.7**	61.1	85.9	74.1	74.8	**64.8**	82.7	75.5	**76.5**
node2vec**	**64.8**	81.8	77.5	78.9	**62.0**	85.4	**81.2**	**81.7**	63.6	82.7	**75.6**	**76.5**
Walklets	59.0	**76.5**	95.7	96.5	43.4	77.0	90.7	90.8	**51.1**	74.2	93.9	93.3
Walklets*	**60.3**	73.9	96.1	97.0	**55.2**	**77.9**	90.8	90.8	48.0	**75.0**	93.1	93.6
Walklets**	56.3	74.1	**96.5**	**97.4**	48.8	74.5	**91.5**	**91.7**	42.1	74.7	**94.2**	**93.8**

## Data Availability

Not applicable.
